# Gene set enrichment analysis provides insight into novel signalling pathways in breast cancer stem cells

**DOI:** 10.1038/sj.bjc.6605468

**Published:** 2009-12-08

**Authors:** M Murohashi, K Hinohara, M Kuroda, T Isagawa, S Tsuji, S Kobayashi, K Umezawa, A Tojo, H Aburatani, N Gotoh

**Affiliations:** 1 Division of Systems Biomedical Technology, Institute of Medical Science, University of Tokyo, Tokyo, Japan; 2 Department of Pathology, Tokyo Medical University, Tokyo, Japan; 3 Genome Science Division, Research Center of Advanced Science and Technology, University of Tokyo, Tokyo, Japan; 4 Division of Molecular Therapy, Institute of Medical Science, University of Tokyo, Tokyo, Japan; 5 Department of Applied Chemistry, Faculty of Science and Technology, Keio University, Yokohama, Japan

**Keywords:** tumour-initiating cells, NF-*κ*B, CD24, CD44, gene expression profiling, DHMEQ

## Abstract

**Background:**

Tumour-initiating cells (TICs) or cancer stem cells can exist as a small population in malignant tissues. The signalling pathways activated in TICs that contribute to tumourigenesis are not fully understood.

**Methods:**

Several breast cancer cell lines were sorted with CD24 and CD44, known markers for enrichment of breast cancer TICs. Tumourigenesis was analysed using sorted cells and total RNA was subjected to gene expression profiling and gene set enrichment analysis (GSEA).

**Results:**

We showed that several breast cancer cell lines have a small population of CD24^−/low^/CD44^+^ cells in which TICs may be enriched, and confirmed the properties of TICs in a xenograft model. GSEA revealed that CD24^−/low^/CD44^+^ cell populations are enriched for genes involved in transforming growth factor-*β*, tumour necrosis factor, and interferon response pathways. Moreover, we found the presence of nuclear factor-*κ*B (NF-*κ*B) activity in CD24^−/low^/CD44^+^ cells, which was previously unrecognised. In addition, NF-*κ*B inhibitor dehydroxymethylepoxyquinomicin (DHMEQ) prevented tumourigenesis of CD24^−/low^/CD44^+^ cells *in vivo*.

**Conclusion:**

Our findings suggest that signalling pathways identified using GSEA help to identify molecular targets and biomarkers for TIC-like cells.

Accumulating evidence suggests that tumour-initiating cells (TICs) or cancer stem cells—which make up only a small proportion of heterogeneous tumour cells—possess a greater ability to maintain tumour formation than other tumour cell types. It has been proposed that TICs have characteristics in common with normal stem cells from tumour-prone tissue (Ailles and Weissman, 2007). For instance, TICs can self-renew and simultaneously produce differentiated daughter cells that proliferate strongly until they reach their final differentiated state. Apparent differences also exist between TICs and normal stem cells. The latter are maintained under tight homoeostatic regulation and are passively protected in the surrounding microenvironment or stem cell niche in adult tissues. However, the former may actively contribute to tumour formation. Although the concept of TICs greatly influences cancer biology and evokes a reconsideration of cancer treatment, the molecular mechanisms involved in the contribution of TICs to tumourigenesis remain obscure.

In human breast cancers, a population characterised by the expression of cell-surface markers, CD24^−/low^/CD44^high^, was reported to be highly enriched in TICs, compared with populations of CD24^high^/CD44^high^ cells (Al-Hajj *et al*, 2003; Mani *et al*, 2008). Two gene-expression profiling studies, comparing CD24^−/low^/CD44^+^ cell populations with other populations in primary breast cancer cells or in normal tissue, presented the CD24^−/low^/CD44^+^ cell population-derived different signatures that seemed to predict poorer prognosis (Liu *et al*, 2007; Shipitsin *et al*, 2007). One study showed that transforming growth factor (TGF)-*β* pathways seem to be activated in these cells (Shipitsin *et al*, 2007). It was subsequently reported that TGF-*β* induced the epithelial–mesenchymal transition (EMT) in mammary glands and stem-like cells in both normal mammary epithelial cells and breast cancer cells (Mani *et al*, 2008). Because TGF-*β* signalling can have positive or negative effects on tumourigenesis, additional signalling may still be needed to stimulate tumourigenesis.

Nuclear factor-*κ*B (NF-*κ*B) is a transcription factor complex and is typically a heterodimer of p50, p52, p65 (RelA), RelB, and c-Rel. It is usually inactive and bound to I*κ*B, an inhibitory protein, in the cytoplasm. Upon stimulation with signals such as tumour necrosis factor (TNF) or interferon (INF), I*κ*B is first phosphorylated, then ubiquitinated, and finally degraded. Released NF-*κ*B translocates to the nucleus and binds to the *κ*B sequence, wherein it promotes the transcription of various genes, including inflammatory cytokines. Nuclear factor-*κ*B is involved in inflammation, angiogenesis, inhibition of apoptosis, and tumourigenesis (Karin *et al*, 2002; Huber *et al*, 2004; Tabruyn and Griffioen, 2008).

Gene set enrichment analysis (GSEA) is a recently developed analytical method of gene-expression profiling. The results are easier to interpret biologically, and the method is more accurate and robust than individual gene analysis methods, such as fold change analysis of expression levels (Subramanian *et al*, 2005).

In this study, we effectively used GSEA to comprehensively analyse signalling pathways in TICs using breast cancer cell lines. As a result, we identified multiple signalling pathways potentially activated in TIC-like cells, including both known and unknown pathways. We found activity of NF-*κ*B, which was previously unrecognised, in TIC-like cells. Therefore, it is possible that the signalling pathways identified using GSEA help to identify novel candidates of molecular targets that have important roles in tumourigenesis in human breast cancer TICs.

## Materials and methods

### Cell culture

Breast cancer cell lines HCC70, HCC1954, MCF7, SK-BR-3, AU-565, BT-474, T-47D, and MDA-MB-231 were purchased from the American Type Culture Collection (ATCC, Manassas, VA, USA). Cells were cultured according to the manufacturer's instructions.

### FACS

Cells were sorted or analysed after staining with CD24-FITC or CD44-PE antibody (BD Pharmingen, San Jose, CA, USA), and dead cells were eliminated using propidium iodide (Sigma, Saint Louis, MO, USA) and FACS VantageSE (BD Biosciences, Bedford, MA, USA). Data were analysed by FlowJo 7.2.2 Tree Star Inc. Ashland, OR, USA.

### Construction of lentiviral vectors

A third-generation self-inactivating lentiviral transfer vector plasmid with a gene encoding firefly luciferase or d2Venus (Nagai *et al*, 2002) (provided by Dr A Miyawaki, RIKEN, Wako, Japan) under the control of the elongation factor 1-*α* (EF1*α*) promoter was produced using constructs provided by Dr H Miyoshi (RIKEN, Tsukuba, Japan) by standard molecular biological techniques (Miyoshi *et al*, 1999). These vectors also contained the central polypurine track and the woodchuck hepatitis virus post-regulatory element. Viral supernatant was produced by transient transfection of 293T cells with packaging plasmids (pMDLg/p.RRE) and HIV rev expression plasmids (pRSV-rev) and was then pseudotyped with the vesicular stomatitis virus G protein (pMD.G), as previously described (Bai *et al*, 2003). High-titre viral stocks were prepared by ultracentrifugation. The functional titres of these vectors (HIV-EF1*α*-Luciferase, HIV-EF1*α*-d2Venus) were determined by infection of HeLa cells using a real-time PCR method (DNA titre; Sastry *et al*, 2002). All the multiplicity of infection (MOI) values used in our experiments were calculated from DNA titres.

### Transduction of cells with lentiviral vectors

HCC1954 and MCF7 cells were pelleted and incubated with viral supernatant at an MOI of 10 in a 1.5-ml Eppendorf tube. After incubation for 2 h at 37°C in 5% CO_2_, cells were cultured until they were used in *in vivo* experiments. Because HIV-EF1a-d2Venus was used for confirmation of transduction efficiency, HIV-EF1a-Luciferase and HIV-EF1a-d2Venus were infected simultaneously in separate tubes. After more than three passages, the cells were used for FACS analysis or in the xenograft model.

### Xenografts

Six-week-old female NOD/SICD mice were anaesthetised with isoflurane (Abbott Japan, Tokyo, Japan), and then 0.72 mg, 60-day-release *β*-estradiol (E2) pellets (Innovative Research of America, Sarasota, FL, USA) were implanted s.c. on the back of the neck (only in the case of MCF7 implantation). A total of 1 × 10^2^ to 3 × 10^4^ sorted cells were suspended in 1 : 1 volumes of phosphate-buffered saline (PBS)(−)/Matrigel (BD Biosciences) to produce 100 *μ*l of mixture and were then injected into the mammary fat pads. Dehydroxymethylepoxyquinomicin (DHMEQ) was suspended in 0.5% chloromethyl cellulose and administrated by i.p. injections of 100 *μ*l containing 12 mg kg^−1^ thrice a week. Control groups were injected with the same volume of vehicle. All treatments started on day 2 after tumour cell implantation.

Mice were handled according to the guidelines of the Institute of Medical Science, University of Tokyo. The experiments were approved by the committee for animal research at the institution.

### *In vivo* imaging

Mice under anaesthesia were injected i.p. with 150 mg kg^−1^ of luciferin (Promega, Madison, WI, USA) in PBS(−), and images were recorded by the IVIS Imaging System (Xenogen, Hopkington, MA, USA) 5 min after the injection. The bioluminescence images were quantified by Living Image software (Xenogen). Observations by IVIS were continued once a week, immediately after the injection, up to 4 weeks. In DHMEQ treatment, tumour growth was monitored by luciferase activity twice a week, for up to 32 days.

### Histology analysis

Tumours from xenograft cells were fixed in 10% neutralised buffered formalin, embedded in paraffin, and then stained with haematoxylin–eosin (HE).

### Microarray analysis

For microarray analysis, 1% of the entire population of the HCC1954, MCF7, or HCC70 cell line, belonging to CD24^−^CD44^+^, was purified on the basis of the lowest expression levels of CD24. In addition ten percent of the entire cell population of each cell line, belonging to CD24^+^/CD44^+^, was purified as the control population (CD24^+^). There was no significant difference in tumourigenicity, whether we considered 1 or 10% of the entire CD24^−/low^/CD44^+^ population as the TIC population. Microarray analyses were performed as previously described (Morikawa *et al*, 2007). Briefly, total RNA was isolated from samples using TRIzol (Invitrogen, Carlsbad, CA, USA) according to the manufacturer's instructions. Six samples were analysed on an Affymetrix high-density oligonucleotide array, Human Genome U133 Plus 2.0. Output images were processed by the MAS5 algorithm and globally scaled to a target intensity of 100. To identify gene-signature-based differences between CD24^−/low^/CD44^+^ and CD24^+^/CD44^+^ populations, we performed GSEA (Subramanian *et al*, 2005). All probe sets were pre-ranked using the ratio of the geometric mean of each group's expression values; thereafter, the ordered probe set list was used as the GSEA input. The detailed GSEA parameters are as follows: the number of permutations is 2500, and the permutation type is configured to the gene set to avoid the potential problem of a small sample size.

### Quantification of NF-*κ*B activity

Nuclear extracts were prepared with a Nuclear Extract Kit according to the manufacturer's instructions (Active Motif, Carlsbad, CA, USA). Briefly, CD24^−/low^/CD44^+^ and CD24^+^/CD44^+^ populations were sorted from the bulk of HCC1954 or MCF7 cells and isolated from the nuclear extracts. NF-*κ*B p65 activity was measured with a TransAM NF-*κ*B p65 Transcription Factor Assay kit (Active Motif) according to the manufacturer's instructions. Four sets of nuclear extracts from each population were prepared, and 20 *μ*g of each extract was used for the p65 NF-*κ*B activity assay.

## Results

### TIC populations in breast cancer cell lines

**Figure 1:**
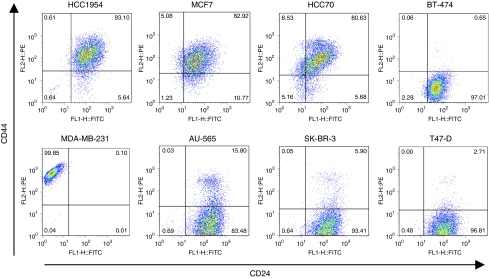
CD24 and CD44 expression patterns in breast cancer cell lines. Expression patterns of CD24 and CD44 in breast cancer cell lines (HCC1954, MCF7, HCC70, BT-474, MDA-MB-231, AU-565, SK-BR-3, and T-47D) were analysed by FACS. Anti-CD24 antibody labelled with FITC and anti-CD44 antibody labelled with PE were applied to the analysis. Gates are based on the isotype control corresponding to each cell line.

To examine whether CD24^−/low^/CD44^+^ cell populations exist in various types of breast cancer cell lines, we analysed the expression of these surface markers in eight breast cancer cell lines by FACS analysis (Figure 1). We found that each cell line had various expression levels of CD24 and CD44. HCC1954, MCF7, HCC70, and MDA-MB-231 cells had relatively high percentages of CD44^+^ cell populations, whereas BT-474, AU-565, SK-BR-3, and T47-D cells showed low CD44 expression levels. HCC1954, MCF7, and HCC70 cells had a small population of CD24^−/low^/CD44^+^ cells. This situation might be similar to that in early-stage breast cancer tissues in which the TIC population is assumed to be small. To determine the hierarchical organisation of breast cancer cell lines, we analysed the tumourigenic potential of the CD24^−/low^/CD44^+^ and CD24^+^/CD44^+^ cell populations of HCC1954 and MCF7 cell lines.

### Tumourigenicity of CD24^−/low^/CD44^+^ cell populations in breast cancer cell lines

The *in vivo* tumourigenicity assay is the gold standard for identifying TICs (Clarke *et al*, 2006). Tumourigenicity of TICs has been examined by using NOD/SCID mouse and measuring palpable tumours. To improve the quality of the quantitative results, we used *in vivo* bioluminescence imaging (IVIS) to measure tumour growth. We first transduced cells with a lentiviral vector encoding luciferase or d2Venus (an improved version of yellow fluorescent protein) cDNA. We measured transduction efficiency by expression levels of d2Venus using FACS. As shown in Supplementary Figure 1, high transduction efficiency was obtained in each cell line: 92.60 and 99.29% for HCC1954 and MCF7 cells, respectively. Next, we transduced a lentiviral vector expressing luciferase into these cells. Because we used similar MOI levels for transduction of the lentiviral vectors expressing luciferase and d2Venus, we expected similar levels of luciferase expression in each cell line. These were designated HCC1954-Luc or MCF7-Luc. Cells in CD24^−/low^/CD44^+^ populations were considered to be enriched for TICs, and CD24^+^CD44^+^ populations were used as controls. We compared the expression levels of luciferase in both cell populations and confirmed that there were no significant differences (Supplementary Figure 2).

**Figure 2:**
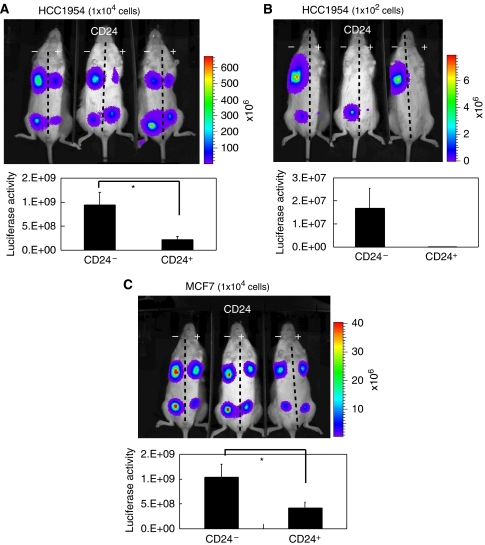
Luciferase activities of CD24^−/low^/CD44^+^ cells in NOD/SCID mice. HCC1954 and MCF7 cells expressing luciferase were sorted by FACS. Ten per cent of the entire population, belonging to CD24^−/low^ CD44^+^, was selected as the TIC population (CD24^−^). Ten per cent of the whole population, belonging to CD24^+^/CD44^+^, was selected as the control (CD24^+^). We transplanted both populations of 1 × 10^4^ HCC1954 cells (**A**), 1 × 10^2^ HCC1954 cells (**B**) of 1 × 10^4^ CF7 cells (**C**) in mammary fat pads of NOD/SCID mice. Luciferase activities were captured by IVIS after 4 weeks (upper panels). Luciferase activities in implanted sites were quantified (*n*=6) (lower graphs). Results are represented as mean±s.d. ^*^P<0.05 (Student's *t*-test).

Cells were implanted into mammary fat pads of NOD/SCID mice and tumour growth was measured by quantifying luciferase activity using the IVIS Imaging System. A total of 10 000 HCC1954-Luc and MCF7-Luc cells of both populations were implanted (Figure 2A and C). After 4 weeks, the analysis of luciferase activity indicated that cells in the CD24^−/low^/CD44^+^ populations of HCC1954-Luc and MCF7-Luc generated significantly larger tumours than the control populations (*P*<0.05). Moreover, when we transplanted both populations of 1 × 10^2^ HCC1954-Luc, we found that tumours were generated only by the CD24^−/low^/CD44^+^ population (*n*=6) (Figure 2B).

These results indicate that CD24^−/low^/CD44^+^ populations in breast cancer cell lines have higher tumourigenicity than control populations. It is therefore likely that CD24^−/low^/CD44^+^ cells in breast cancer cell lines may behave in a manner similar to TICs.

We also examined the histology of tumours derived from HCC1954-Luc cells from both populations and from an unsorted population when 1 × 10^4^ cells of each population were implanted. HE staining revealed that tumours derived from CD24^−/low^/CD44^+^ and unsorted cells showed a similar histology, namely, exclusively invasive patterns with a variety of morphologies and the stromal component (Supplementary Figure 3). In contrast, tumours derived from control cells showed both invasive and differentiated patterns associated with a smaller stromal component than CD24^−/low^/CD44^+^ or unsorted cells.

### Gene-expression profiling and GSEA for the identification of pathways and key effectors in CD24^−/low^/CD44^+^ cells

**Figure 3:**
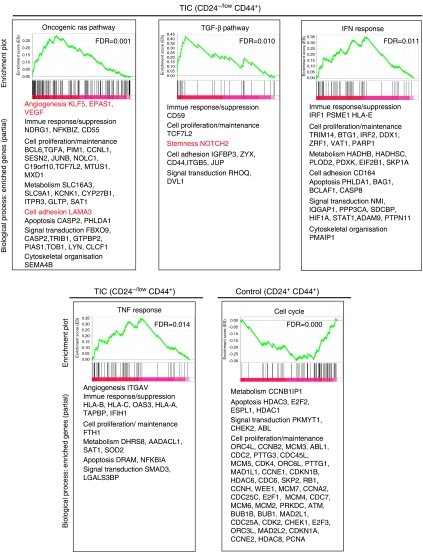
Gene set enrichment analysis. DNA microarray analyses were performed to compare TIC and control populations of HCC70, HCC1954, and MCF7. One per cent of the whole population of each cell line, belonging to CD24^−^/CD44^+^, purified on the basis of the lowest expression levels of CD24, was selected as the TIC population. Ten per cent of the whole population, belonging to CD24^+^/CD44^+^, was purified for the control. Microarray data were ranked using the geometric mean of the expression ratios between the TIC and control populations from the three cell lines, and GSEA was then applied. GSEA-extracted representative pathways containing genes enriched in the TIC or control populations are shown. In the original GSEA data sets, the oncogenic Ras pathway is depicted as RAS_ONCOGENIC_SIGNATURE, the TGF-*β* pathway is depicted as TGFBETA_ EARLY_UP, the IFN response is depicted as IFN_ANY_UP, and the TNF response pathway is depicted as SANA_TNFA_ENDOTHELIAL_UP.

To identify expressed genes that were highly enriched in CD24^−/low^/CD44^+^ and control cells, we performed DNA microarray analysis using HCC1954, MCF7, and HCC70 cell lines that have small populations of CD24^−^/CD44^+^ cells. To select cell populations strictly, we used only CD24^−^/CD44^+^ cell populations that accounted for approximately 1% of the entire population. As control, we used CD24^+^/CD44^+^ cell populations that comprised approximately 10% of the entire population. Microarray data were ranked by the expression ratio between the geometric mean of the CD24^−/low^/CD44^+^:CD24^+^/CD44^+^ populations from the three cell lines (Supplementary Table). We then applied GSEA (Subramanian *et al*, 2005). Our results showed that gene sets involving TGF-*β* pathways and oncogeneic Ras pathways were upregulated in CD24^−/low−^/CD44^+^ populations (Figure 3). Moreover, we found that both *TNF* and IFN response gene signatures were markedly enriched in CD24^−/low−^/CD44^+^ populations.

With regard to individual genes, gene-ontology-based classification revealed that genes involved in ‘stemness’, cell proliferation/maintenance, cell adhesion, cell motility, invasion, angiogenesis, growth factor/cytokine, immune response/suppression, and metabolism were highly represented in CD24^−/low−^/CD44^+^ cells compared with control cell populations. All these genes may contribute to oncogenesis. For example, from the GSEA results, we found *Notch2*, a ‘stemness’-related gene, in the TGF-*β* pathway; *LAMA3*, a cell invasion- or adhesion-related gene, in the oncogenic Ras pathway; and *KLF5*, *EPAS1,* and *VEGF*, angiogenesis-related genes, in the oncogenic Ras pathway (Figure 3, in red). Conversely, GSEA revealed that genes highly expressed in control populations correlated with several cell-cycle-associated gene sets, which have large numbers of cell proliferation/maintenance-related genes.

**Figure 4:**
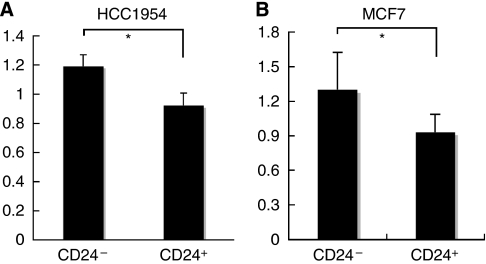
Activity of NF-*κ*B. Quantification of NF-*κ*B activity in CD24^−^/CD44^+^ and CD24^+^/CD44^+^ populations sorted from HCC1954 (**A**) and MCF7 cells (**B**). The data (mean±s.d.) are representative of three experiments. ^*^P<0.05.

One of the important effector molecules common to both TNF and IFN response pathways is NF-*κ*B. We quantified NF-*κ*B activities in nuclear extracts of CD24^−/low^/CD44^+^ and control populations that were sorted by FACS analysis. We found that the activity of NF-*κ*B was significantly higher in CD24^−/low^/CD44^+^ than in CD24^+^/CD44^+^ populations (Figure 4).

The TNF or IFN response pathway is involved in the expression of many inflammatory cytokines/chemokines. Vascular endothelial growth factor A, interleukin 8, and chemokine (C-C motif) ligand 5 are among the inflammatory cytokines/chemokines associated with stroma-like activities (Shono *et al*, 1996; Moriuchi *et al*, 1997; Yoshida *et al*, 1997; Cho *et al*, 2007). Among the highly ranked genes, we also noticed Toll-like receptor 1, another upstream activator for NF-*κ*B, and stromal cell-derived factor 2-like 1, which is reported to be upregulated through EMT, an important biological output of the TGF-*β* pathway (Massagué, 2008; Sarrio *et al*, 2008; Rakoff-Nahoum and Medzhitov, 2009). We measured the expression levels of these genes by quantitative RT-PCR and confirmed that they were expressed at significantly higher levels in CD24^−/low−^/CD44^+^ populations compared with control cells (Supplementary Figure 4).

### Decreased tumourigenesis in CD24^−/low^/CD44^+^ populations after treatment with DHMEQ, a specific inhibitor for NF-*κ*B

**Figure 5:**
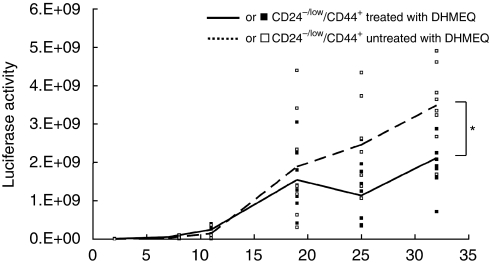
Effects on tumourigenesis after treatment with NF-*κ*B inhibitor. Tumour growth of CD24^−/low^/CD44^+^ populations of HCC1954 cells treated with NF-*κ*B inhibitor DHMEQ was measured by luciferase activity (*n*=8). Averages of luciferase activity are indicated by lines. ^*^P<0.05.

We next examined the role of NF-*κ*B activity in tumourigenesis using a mouse model. TICs are believed to be important at the beginning of tumourigenesis or in its recurrence; therefore, we analysed tumourigenesis at early points from relatively small numbers of TIC-like cells. We transplanted 10^4^ cells of CD24^−/low^/CD44^+^ populations into NOD/SCID mice, as shown in Figure 2, and treated them with DHMEQ, a specific inhibitor for NF-*κ*B (Umezawa, 2006; Supplementary Results; Supplementary Figures 5 and 6). To analyse the effects occurring during the course of tumourigenesis, we began inhibitor treatment 2 days after transplantation. We monitored tumour formation by *in vivo* imaging and found that the luciferase activities of the tumours derived from CD24^−/low^/CD44^+^ cell populations treated with DHMEQ were significantly decreased compared with that of untreated cell-derived tumours (Figure 5). This finding suggests that NF-*κ*B functions as a key effector of tumourigenesis derived from TIC-like cells.

## Discussion

In this study, we established a mouse model that may recapitulate part of the tumourigenic process in TICs, using breast cancer cell lines. We showed that cells derived from CD24^−/low^/CD44^+^ populations resulted in tumours larger than those of CD24^+^/CD44^+^ control populations. Importantly, when as few as 100 cells were implanted, only CD24^−/low^/CD44^+^ populations gave rise to tumours (Figure 2B). This is an important criterion for TICs (Clarke *et al*, 2006). Therefore, the CD24^−/low^/CD44^+^ populations in cell lines may be enriched with TIC-like cells. Our results revealed heterogeneity in cell populations divided into TIC-like cells and other cells. Consistent with our data, it has been recently reported that other cell lines also have TIC-like cell populations (Fillmore and Kuperwasser, 2008). Therefore, it is reasonable to assume that several breast cancer cell lines are heterogeneous and that they have distinct cell populations: TIC-like cells and other cells, with both cell types preserving the characteristics of TICs and other cells in primary cancer tissues to some extent.

Moreover, we labelled cells with a luciferase reporter gene and established a monitoring system of tumourigenesis in the fat pads of NOD/SCID mice by sensitive and quantitative *in vivo* imaging that can detect as few as 100 cells. Because TICs are thought to be important in tumourigenesis during transition from the pre-malignant stage to the malignant stage or during recurrence with a few tumour cells, this model should be useful for monitoring tumourigenesis at these stages. Indeed, we were able to validate candidate targets in TICs using inhibitors for NF-*κ*B in our model (Figure 5).

Gene-expression profiling combined with GSEA showed that several signalling pathways and genes are involved in CD24^−/low^/CD44^+^ TIC-like cells compared with CD24^+^/CD44^+^ control populations. Gene Ontology Classification revealed that a variety of genes may represent malignant characteristics of CD24^−/low^/CD44^+^ TIC-like cells. There is an overlap with those genes found in a previous report using cells from CD24^−/low^/CD44^+^ and CD24^+^/CD44^+^ populations derived from normal breast and primary breast cancer tissues. Some genes involved in the TGF-*β* pathway were enriched in CD24^−/low^/CD44^+^ populations, consistent with the previous report (Shipitsin *et al*, 2007). Importantly, we found genes associated with oncogenic Ras pathways, as well as with TNF and IFN response pathways, as novel gene sets in CD24^−/low^/CD44^+^ populations. It is likely that genes involved in the oncogenic Ras pathway, the TNF response pathway, and the IFN response pathway are specifically represented in TICs but not in normal stem cells.

The TGF-*β* pathway inhibits tumourigenesis when it is the only pathway activated in cells (Massagué, 2008). However, in the malignant state, the TGF-*β* pathway cooperates with other pathways to facilitate tumourigenesis. It has been reported that the oncogenic Ras pathway, inflammatory responses, and activation of NF-*κ*B are such cooperative pathways (Huber *et al*, 2004).

These findings suggest that TICs are more malignant than other cells in cancer. We also cannot rule out the possibility that cell lines may have additional characteristics that differ from those of primary cells. For example, cell-cycle-related gene sets found to be enriched in control cells may somehow reflect *in vitro* culture adaptations (Figure 3). The enhanced cell-cycling activity might allow control cells to grow *in vivo* after implantation; whereas control cells derived from primary tissues rarely generate tumours *in vivo* (Figure 2).

We showed that activity of NF-*κ*B is higher in TIC-like cells than in control cells. Moreover, DHMEQ, a highly specific inhibitor for NF-*κ*B, suppressed tumourigenesis in the TIC-like cells in our mouse model. This was assessed following treatment that occurred soon after transplantation. Thus, NF-*κ*B could be a promising target for treatment of early stages of breast cancer and for the prevention of recurrence.

Taken together, our findings raise an intriguing possibility: TICs behave in a manner similar to CAFs and can actively generate and maintain the cancer stem cell niche, in which NF-*κ*B functions as the main effector that can induce many secretory proteins, including cytokines and chemokines. Future studies should focus on the extensive evaluation of our model by using clinical samples of breast cancer.

## Supplementary Material

Supplementary Table

Supplementary Results

## References

[bib1] Ailles LE, Weissman IL (2007) Cancer stem cells in solid tumors. Curr Opin Biotechnol 18: 460–466 18023337 10.1016/j.copbio.2007.10.007

[bib2] Al-Hajj M, Wicha MS, Benito-Hernandez A, Morrison SJ, Clarke MF (2003) Prospective identification of tumorigenic breast cancer cells. Proc Natl Acad Sci USA 100: 3983–3988 12629218 10.1073/pnas.0530291100 PMC153034

[bib3] Bai Y, Soda Y, Izawa K, Tanabe T, Kang X, Tojo A, Hoshino H, Miyoshi H, Asano S, Tani K (2003) Effective transduction and stable transgene expression in human blood cells by a third-generation lentiviral vector. Gene Therapy 10: 1446–1457 12900759 10.1038/sj.gt.3302026

[bib4] Cho ML, Ju JH, Kim HR, Oh HJ, Kang CM, Jhun JY, Lee SY, Park MK, Min JK, Park SH, Lee SH, Kim HY (2007) Toll-like receptor 2 ligand mediates the upregulation of angiogenic factor, vascular endothelial growth factor and interleukin-8/CXCL8 in human rheumatoid synovial fibroblasts. Immunol Lett 108: 121–128 17182109 10.1016/j.imlet.2006.11.005

[bib5] Clarke MF, Dick JE, Dirks PB, Eaves CJ, Jamieson CH, Jones DL, Visvader J, Weissman IL, Wahl GM (2006) Cancer stem cells—perspectives on current status and future directions: AACR Workshop on cancer stem cells. Cancer Res 66: 9339–9344 16990346 10.1158/0008-5472.CAN-06-3126

[bib6] Fillmore CM, Kuperwasser C (2008) Human breast cancer cell lines contain stem-like cells that self-renew, give rise to phenotypically diverse progeny and survive chemotherapy. Breast Cancer Res 10: R25 18366788 10.1186/bcr1982 PMC2397524

[bib7] Huber MA, Beug H, Wirth T (2004) Epithelial–mesenchymal transition: NF-kappaB takes center stage. Cell Cycle 3: 1477–1480 15539952 10.4161/cc.3.12.1280

[bib8] Karin M, Cao Y, Greten FR, Li ZW (2002) NF-kappaB in cancer: from innocent bystander to major culprit. Nat Rev Cancer 2: 301–310 12001991 10.1038/nrc780

[bib9] Liu R, Wang X, Chen GY, Dalerba P, Gurney A, Hoey T, Sherlock G, Lewicki J, Shedden K, Clarke MF (2007) The prognostic role of a gene signature from tumorigenic breast-cancer cells. N Engl J Med 356: 217–226 17229949 10.1056/NEJMoa063994

[bib10] Mani SA, Guo W, Liao MJ, Eaton EN, Ayyanan A, Zhou AY, Brooks M, Reinhard F, Zhang CC, Shipitsin M, Campbell LL, Polyak K, Brisken C, Yang J, Weinberg RA (2008) The epithelial–mesenchymal transition generates cells with properties of stem cells. Cell 133: 704–715 18485877 10.1016/j.cell.2008.03.027 PMC2728032

[bib11] Massagué J (2008) TGF*β* in Cancer. Cell 134: 215–230 18662538 10.1016/j.cell.2008.07.001 PMC3512574

[bib12] Miyoshi H, Smith KA, Mosier DE, Verma IM, Torbett BE (1999) Transduction of human CD34^+^ cells that mediate long-term engraftment of NOD/SCID mice by HIV vectors. Science 283: 682–686 9924027 10.1126/science.283.5402.682

[bib13] Morikawa T, Sugiyama A, Kume H, Ota S, Kashima T, Tomita K, Kitamura T, Kodama T, Fukayama M, Aburatani H (2007) Identification of Toll-like receptor 3 as a potential therapeutic target in clear cell renal cell carcinoma. Clin Cancer Res 13: 5703–5709 17908959 10.1158/1078-0432.CCR-07-0603

[bib14] Moriuchi H, Moriuchi M, Fauci AS (1997) Nuclear factor-kappa B potently up-regulates the promoter activity of RANTES, a chemokine that blocks HIV infection. J Immunol 158: 3483–3491 9120310

[bib15] Nagai T, Ibata K, Park ES, Kubota M, Mikoshiba K, Miyawaki A (2002) A variant of yellow fluorescent protein with fast and efficient maturation for cell-biological applications. Nat Biotechnol 20: 87–90 11753368 10.1038/nbt0102-87

[bib16] Rakoff-Nahoum S, Medzhitov R (2009) Toll-like receptors and cancer. Nat Rev Cancer 9: 57–63 19052556 10.1038/nrc2541

[bib17] Sarrio D, Rodriguez-Pinilla SM, Hardisson D, Cano A, Moreno-Bueno G, Palacios J (2008) Epithelial–mesenchymal transition in breast cancer relates to the basal-like phenotype. Cancer Res 68: 989–997 18281472 10.1158/0008-5472.CAN-07-2017

[bib18] Sastry L, Johnson T, Hobson MJ, Smucker B, Cornetta K (2002) Titering lentiviral vectors: comparison of DNA, RNA and marker expression methods. Gene Therapy 9: 1155–1162 12170379 10.1038/sj.gt.3301731

[bib19] Shipitsin M, Campbell LL, Argani P, Weremowicz S, Bloushtain-Qimron N, Yao J, Nikolskaya T, Serebryiskaya T, Beroukhim R, Hu M, Halushka MK, Sukumar S, Parker LM, Anderson KS, Harris LN, Garber JE, Richardson AL, Schnitt SJ, Nikolsky Y, Gelman RS, Polyak K (2007) Molecular definition of breast tumor heterogeneity. Cancer Cell 11: 259–273 17349583 10.1016/j.ccr.2007.01.013

[bib20] Shono T, Ono M, Izumi H, Jimi SI, Matsushima K, Okamoto T, Kohno K, Kuwano M (1996) Involvement of the transcription factor NF-kappaB in tubular morphogenesis of human microvascular endothelial cells by oxidative stress. Mol Cell Biol 16: 4231–4239 8754823 10.1128/mcb.16.8.4231 PMC231421

[bib21] Subramanian A, Tamayo P, Mootha VK, Mukherjee S, Ebert BL, Gillette MA, Paulovich A, Pomeroy SL, Golub TR, Lander ES, Mesirov JP (2005) Gene set enrichment analysis: a knowledge-based approach for interpreting genome-wide expression profiles. Proc Natl Acad Sci USA 102: 15545–15550 16199517 10.1073/pnas.0506580102 PMC1239896

[bib22] Tabruyn SP, Griffioen AW (2008) NF-kappa B: a new player in angiostatic therapy. Angiogenesis 11: 101–106 18283548 10.1007/s10456-008-9094-4 PMC2268731

[bib23] Umezawa K (2006) Inhibition of tumor growth by NF-kappaB inhibitors. Cancer Sci 97: 990–995 16925581 10.1111/j.1349-7006.2006.00285.x PMC11158475

[bib24] Yoshida S, Ono M, Shono T, Izumi H, Ishibashi T, Suzuki H, Kuwano M (1997) Involvement of interleukin-8, vascular endothelial growth factor, and basic fibroblast growth factor in tumor necrosis factor alpha-dependent angiogenesis. Mol Cell Biol 17: 4015–4023 9199336 10.1128/mcb.17.7.4015 PMC232254

