# Supplementary material for: Gene set enrichment analysis provides insight into novel signalling pathways in breast cancer stem cells
Source: Br J Cancer. 2009 Dec 8;102(1):206–12. doi: 10.1038/sj.bjc.6605468 (PMC2813736; doi:10.1038/sj.bjc.6605468)
Supplement: Supplementary Figures 1–6 [file 6605468x1.pdf]

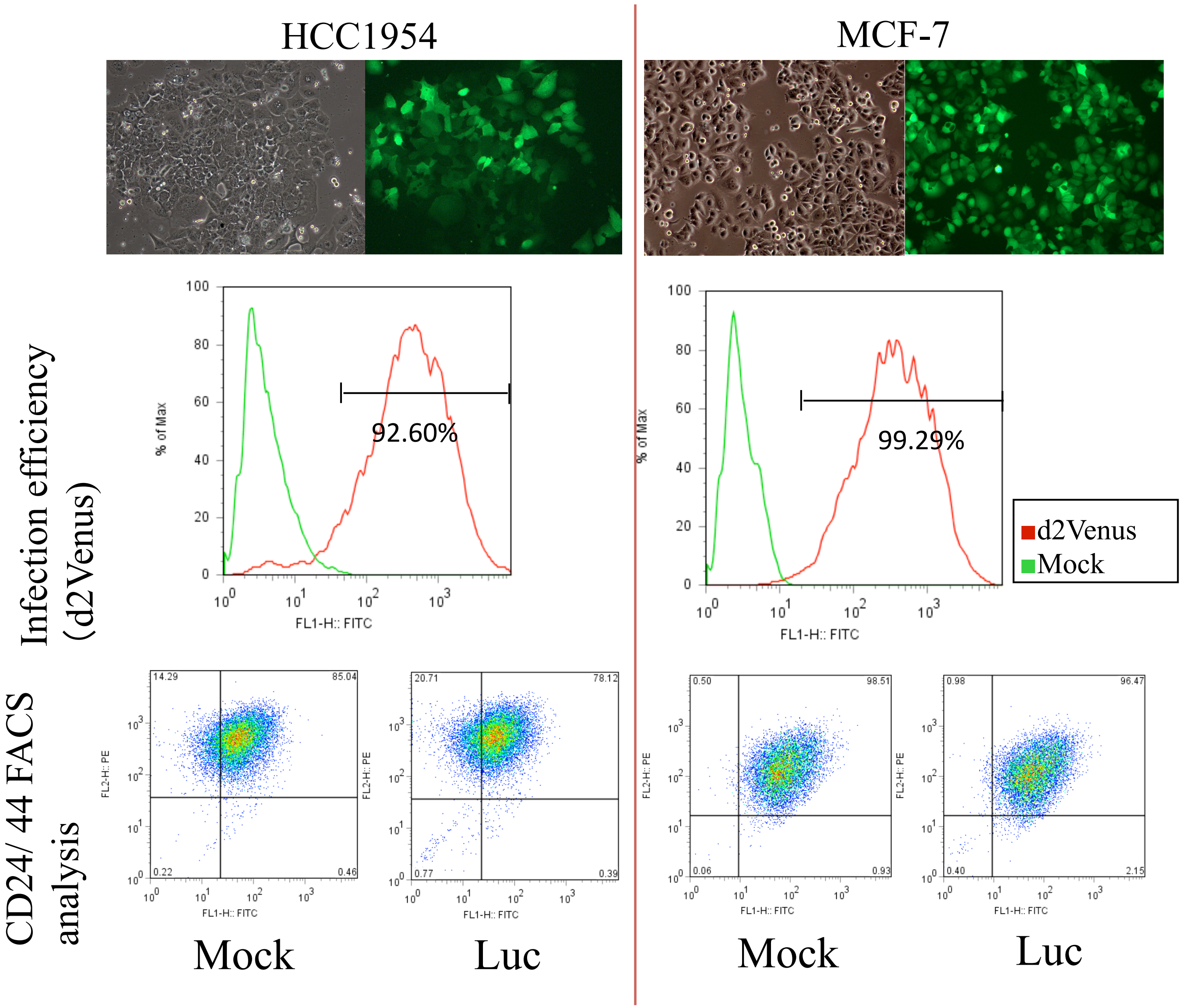

Supplementary Figure 1

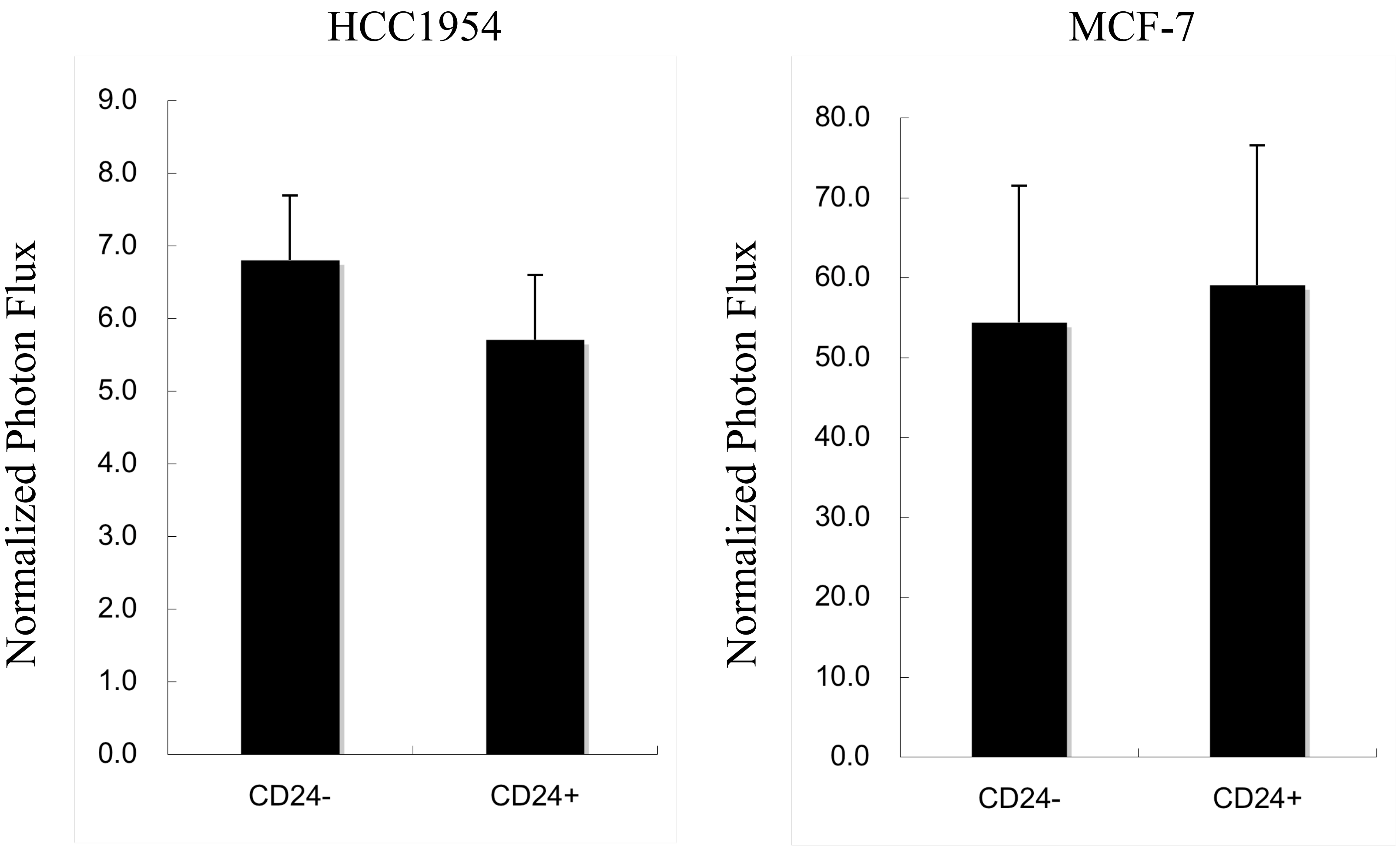

Supplementary Figure 2

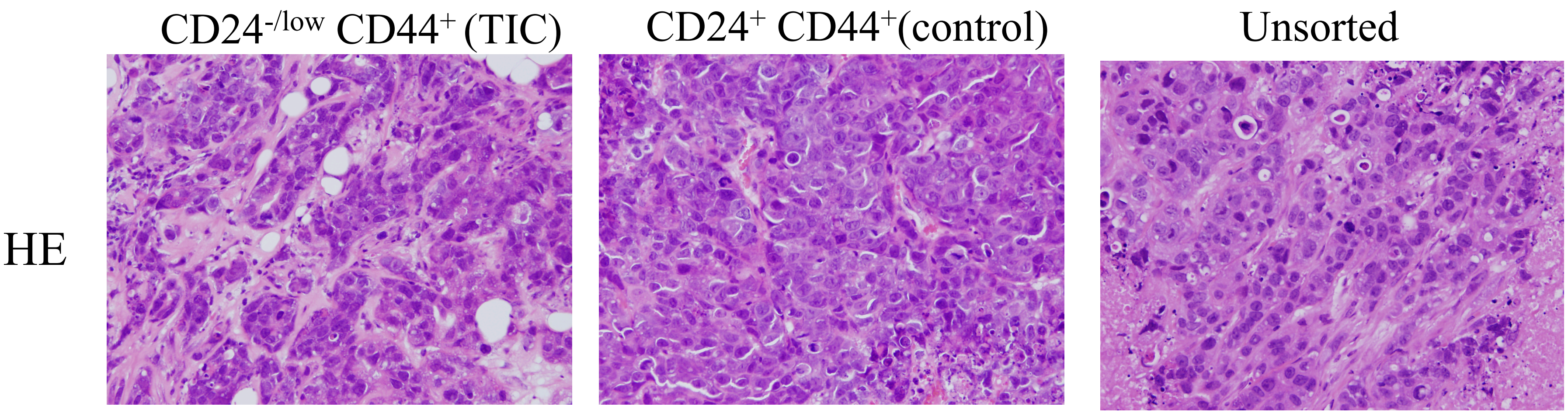

Supplementary Figure 3

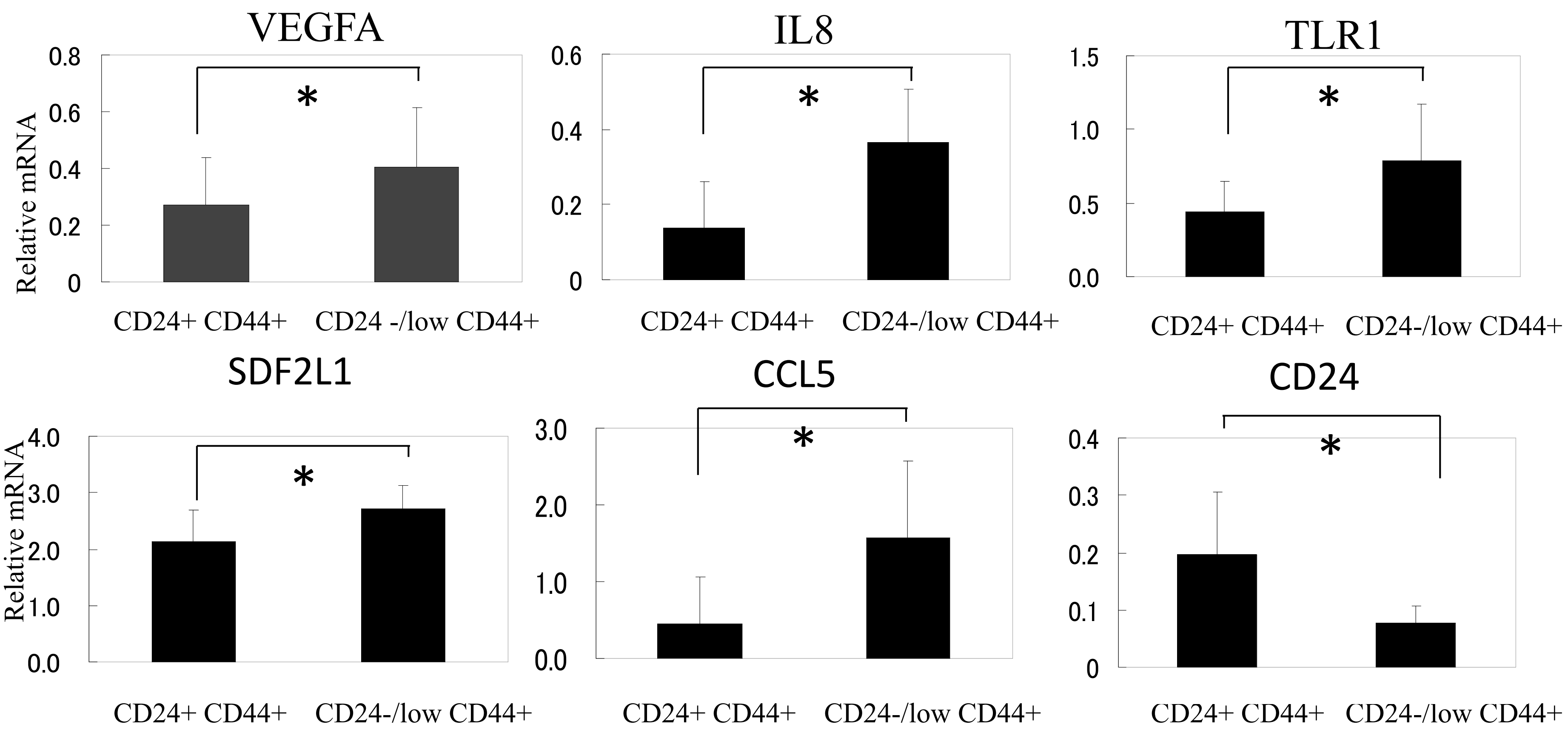

Supplementary Figure 4

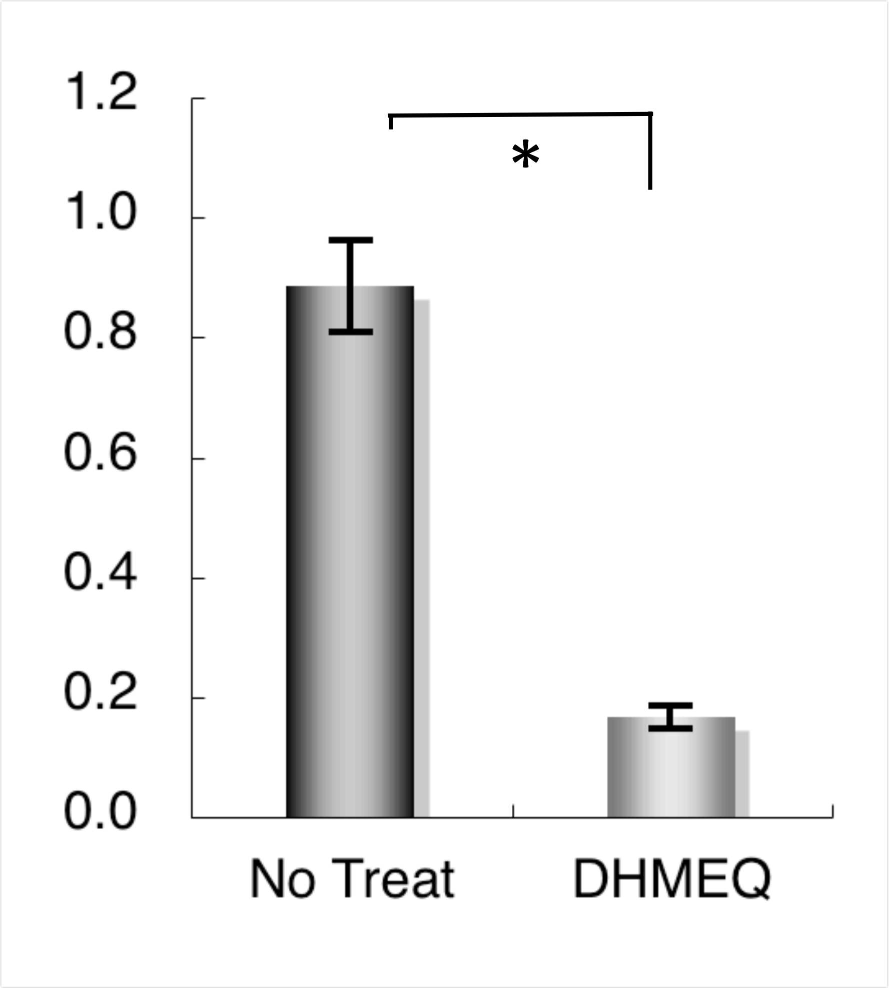

Supplementary Figure 5

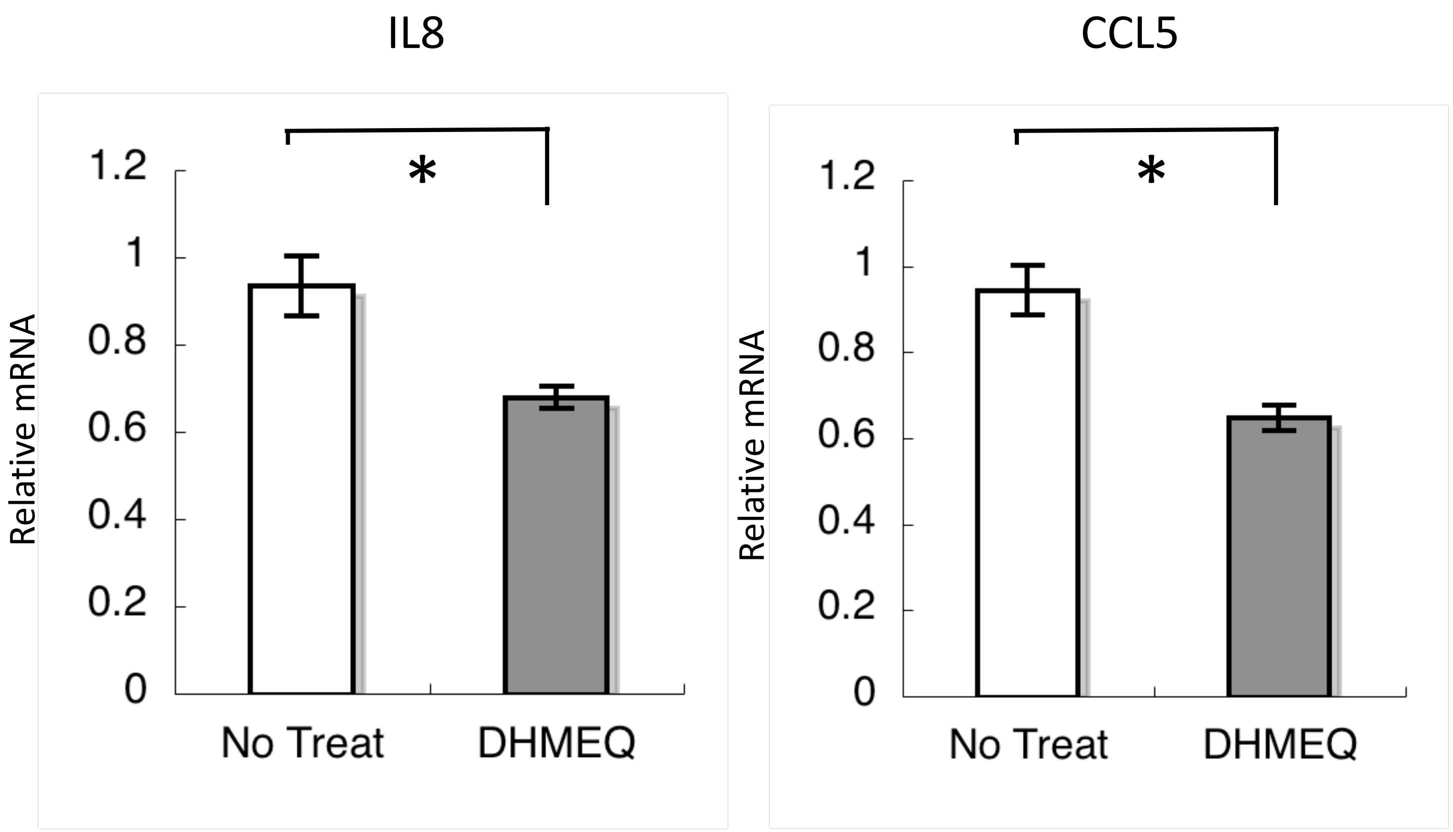

Supplementary Figure 6
