# Supplementary material for: Gene set enrichment analysis provides insight into novel signalling pathways in breast cancer stem cells
Source: Br J Cancer. 2009 Dec 8;102(1):206–12. doi: 10.1038/sj.bjc.6605468 (PMC2813736; doi:10.1038/sj.bjc.6605468)
Supplement: Supplementary Results [file 6605468x3.doc]

**Supplementary matierials and methods**

**Quantitative RT-PCR**

Total RNA was isolated with Isogen (Nippon Gene) according to the manufacturer’s protocol. One microgram of total RNA was used for reverse transcription with a High Capacity cDNA Reverse Transcription Kit (Applied Biosystems). Quantitative RT-PCR was performed by Step ONE plus (Applied Biosystems) within 40 cycles with Taqman probes for β-Actin (Hs99999903_m1), VEGFA (Hs00900054_m1), IL8 (Hs00174103_m1), TLR1 (Hs00413978_m1), SDF2L1 (Hs00222786_m1), CCL5 (Hs00174575_m1) and CD24 (Hs00273561_s1). Quantifications were based on standard curves.

**DHMEQ treatment on HCC1954 cells**

HCC1954 cells were sorted using CD24-FITC or CD44-PE antibody following a 3 h treatment with 10μg/ml DHMEQ or vehicle. Total RNA or nuclear extracts were prepared from CD24^-/low^/CD44^+^ HCC1954 cells.

**Supplementary results**

**Decreased levels of NF-kB activity in CD24^-/low-^/CD44^+^ cells in association with reduced expression levels of transcripts of the inflammatory cytokines.**

To assess the effects of DHMEQ treatment, we measured NF-kB activity and expression levels of transcripts of the inflammatory cytokines in CD24^-/low-^/CD44^+^ cells after treatment with DHMEQ. We found that NF-kB activity was significantly reduced in DHMEQ-treated CD24^-/low-^/CD44^+^ cells than in untreated cells (Supplementary Figure 5). We also found significantly reduced expression levels of transcripts of IL8 and CCL5 in DHMEQ-treated CD24^-/low-^/CD44^+^ cells than in untreated cells (Supplementary Figure 6). This result is consistent with the notion that IL8 or CCL5 may be regulated by NF-kB activity in TIC cells.

**Supplementary Figure legends**

**Supplementary Figure 1**

**Transduction efficiency of lentiviral vector.**

HCC1954 and MCF7 cells were infected with HIV-EF1α-d2Venus and HIV-EF1a-Luciferase. The transduction efficiency was evaluated using HIV-EF1a-d2Venus infected cells with flow cytometry (middle panels) and green fluorescence (upper panels). HCC1954 and MCF7 cells were stained with CD24-FITC and CD44-PE and analyzed by flow cytometry before and after the infection with HIV-EF1α-Luciferase (lower panels).

**Supplementary Figure 2**

Quantification of the normalized photon flux measured following incubation with D-luciferin of sorted cells (CD24^-/low^/CD44^+^ and CD24^+^/CD44^+^) from HCC1954 and MCF-7 cell lines that were infected with the luciferase vector. To assess the efficiency of the lentivirus infection, 10,000 cells were plated for both cell lines. Comparable levels of luciferase infection were detected for the CD24^-/low^/CD44^+^ and CD24^+^/CD44^+^ populations of the both cell lines.

**Supplementary Figure 3**

**Histology of tumors from HCC1954.**

HE-stained sections of the tumors derived from CD24^-/low^/CD44^+^ cells (TICs), CD24^+^/CD44^+^ (control) cells and unsorted cells. All images were taken at x20 magnification.

**Supplementary Figure 4**

**Quantitative RT-PCR analysis.**

VEGFA, IL8, TLR1, SDF2L1, CCL5 and CD24 (as a control) were examined by qRT-PCR using HCC1954 sorted cell populations. The data (mean ± SD) are representative of six experiments. *p<0.05

**Supplementary Figure 5**

**NF-kB activity in DHMEQ-treated CD24^-/low^/ CD44^+^ cells.**

NFkB activity in CD24^-/low^/ CD44^+^ cells was measured after treatment with DHMEQ or vehicle. The data (mean ± SD) are representative of three experiments. *p<0.05

**Supplementary Figure 6**

**Expression of transcripts of the inflammatory cytokines in DHMEQ-treated CD24^-/low^/ CD44^+^ cells.**

IL8 and CCL5 were examined by qRT-PCR using HCC1954 sorted cell populations after treatment with DHMEQ. The data (mean ± SD) are representative of six experiments. *p<0.05

**Supplementary Table**

The microarray data were ranked by the expression ratio between the geometric mean of the CD24^-/low^/CD44^+^:CD24^+^/CD44^+^ populations from the three cell lines.
